# E-Cigarette Use and Symptoms of Depression and Anxiety Among US Middle and High School Students

**DOI:** 10.5888/pcd22.250186

**Published:** 2025-08-14

**Authors:** Brenna VanFrank, Tonya R. Williams, Iris C. Alcantara, Marci Hertz, Maeh Al-Shawaf, Christina Meyers, Andrenita West

**Affiliations:** 1 Office on Smoking and Health, National Center for Chronic Disease Prevention and Health Promotion, Centers for Disease Control and Prevention, Atlanta, Georgia; 2 Division of Adolescent and School Health, National Center for Chronic Disease Prevention and Health Promotion, Centers for Disease Control and Prevention, Atlanta, Georgia

## Abstract

**Introduction:**

E-cigarettes are the most commonly used tobacco product among youth. Most e-cigarettes contain nicotine, which is highly addictive and can harm the developing brain. Youth e-cigarette use is associated with poor mental health.

**Methods:**

We analyzed self-reported data from the 2024 National Youth Tobacco Survey to describe e-cigarette use and symptoms of depression and anxiety among US middle and high school students.

**Results:**

In 2024, 42.1% of youth who currently used e-cigarettes reported moderate-to-severe symptoms of depression and anxiety compared with 21.0% of youth who never or formerly used e-cigarettes. Among youth who currently used e-cigarettes, those with moderate-to-severe symptoms of depression and anxiety (vs no-to-mild symptoms) more frequently reported symptoms of dependence — wanting to use an e-cigarette within 30 minutes of waking (28.2% vs 15.6%, *P* < .001) and having strong cravings (37.6% vs 22.4%, *P* < .001) — and “feeling anxious, stressed, or depressed” as a reason for first (41.8% vs 18.4%, *P* < .001) and current (51.0% vs 25.2%, *P* < .001) use. Most youth who used e-cigarettes attempted to quit in the past year (69.4%), but over half (58.5%) did not use any quitting resources.

**Conclusion:**

Moderate-to-severe symptoms of depression and anxiety are common among youth who use e-cigarettes. Youth with these symptoms may need additional support to prevent or quit e-cigarette use. Integrating mental health support into comprehensive approaches to tobacco use prevention and cessation, paired with strengthening the foundations of healthy communities for youth, may reduce youth e-cigarette use.

**Summary**

**What is already known about this topic?**

E-cigarettes are the most commonly used tobacco product among youth. Most e-cigarettes contain nicotine, which is addictive and can harm the developing brain.

**What is added by this report?**

In 2024, 42.1% of youth who currently used e-cigarettes reported moderate-to-severe symptoms of depression and anxiety compared with 21.0% of youth who did not use e-cigarettes. Among youth who used e-cigarettes, those with moderate-to-severe symptoms of depression and anxiety (vs no-to-mild symptoms) more frequently reported symptoms of nicotine dependence.

**What are the implications for public health practice?**

Youth with symptoms of poor mental health may need additional support to prevent or quit e-cigarette use. Integrating mental health support into comprehensive approaches for tobacco use prevention and cessation may reduce youth e-cigarette use.

## Introduction

In 2024, 8.1% (2.25 million) of US middle and high school students reported current use of any tobacco product (1). Most tobacco products, including e-cigarettes, contain nicotine, a substance that poses unique dangers to the developing brain (2). Nicotine is highly addictive and can harm areas of the brain that control attention, learning, mood, and impulse control (2). While the prevalence of youth who reported current use of e-cigarettes declined from 7.7% in 2023 to 5.9% in 2024, e-cigarettes remain the most commonly used tobacco product among youth (1).

Youth use of e-cigarettes is associated with poor mental health (2–5). In a 2022–2023 study, youth and young adults with severe or extremely severe levels of depression, anxiety, and stress were more likely to report current use of e-cigarettes than those with “normal levels” (5). Similarly, a 2021 systematic review found associations between e-cigarette use and depression, anxiety, suicidality, externalizing problems (ie, attention-deficit or hyperactivity disorder and conduct disorder), and perceived stress (3). Poor mental health might both be exacerbated by e-cigarette use and predispose youth to use e-cigarettes (2–5).

Despite evidence of an association between e-cigarette use and poor mental health, little is known about e-cigarette use behaviors, such as frequency of use and quitting behaviors, among youth who are experiencing poor mental health. To guide ongoing efforts to prevent tobacco use initiation and facilitate cessation among youth, this report examines e-cigarette use behaviors of students who currently use e-cigarettes by presence and severity of symptoms of depression and anxiety.

## Methods

### Data collection

The 2024 National Youth Tobacco Survey (NYTS) is a cross-sectional, voluntary, school-based, self-administered, internet survey of US middle and high school students (grades 6–12) (1). Data were collected from January 22 through May 22, 2024.

### Measures

We defined current e-cigarette use as e-cigarette use on 1 or more days in the past 30 days and former e-cigarette use as ever having used an e-cigarette, but not in the last 30 days. We combined former and never e-cigarette use into a single category. E-cigarettes were defined for respondents in the survey as “battery powered devices that usually contain a nicotine-based liquid that is vaporized and inhaled.” Respondents were additionally provided a list of example e-cigarette brands and alternative terms for e-cigarettes (eg, “vapes”).

Demographic characteristics included school level (middle or high school), sex, race and ethnicity, and grades in school. E-cigarette use behaviors included measures of dependence, quitting behaviors, use of flavors, and co-use of e-cigarettes and other tobacco products. Definitions of these measures appear in table footnotes.

NYTS assesses symptoms of depression and anxiety using the validated Patient Health Questionnaire-4 (PHQ-4) (6,7). The PHQ-4 scale comprises 4 questions: “During the past two weeks, how often have you been bothered by any of the following problems? 1) Little interest or pleasure in doing things; 2) Feeling down, depressed, or hopeless; 3) Feeling nervous, anxious, or on edge; and 4) Not being able to stop or control worrying.” A composite score is generated based on answers to each of the questions: “not at all” = 0, “several days” = 1, “more than half of the days” = 2, and “nearly every day” = 3. Symptom severity is categorized based on composite score: none (score of 0–2), mild (score of 3–5), moderate (score of 6–8), and severe (score of 9–12) (6,7). For this analysis, we dichotomized these categories (no-to-mild and moderate-to-severe).

Reasons for first and current e-cigarette use were assessed with the questions “Why did you first use [Why do you currently use] an e-cigarette? (Select one or more).” Each question had 13 pre-determined response options including “some other reason” which allowed a write-in response. We determined the top 5 reasons reported for first and current e-cigarette use for both symptom severity groups.

### Analysis

We assessed differences in demographic characteristics and symptoms of depression and anxiety by e-cigarette use (current vs former or never) among all respondents. To assess differences in e-cigarette use behaviors by symptom severity, we limited the analysis to youth who reported current e-cigarette use and completed all PHQ-4 questions. E-cigarette use behaviors and reasons for use were stratified by symptom severity (no-to-mild vs moderate-to-severe).

In 2024, 29,861 students from 283 schools participated in NYTS, yielding an overall response rate of 33.4%. Among respondents, data on e-cigarette use was missing for 0.1% and PHQ-4 responses were missing for 16.1% (33.4% of respondents reporting current e-cigarette use and 14.8% of respondents reporting former or never use). The analytic sample for overall comparisons by e-cigarette use status was 29,501 youth. The analytic sample for comparisons by symptom severity among youth reporting current e-cigarette use was 1,219.

We conducted statistical analyses using SAS 9.4 (version 11.4, Research Triangle Institute) with survey procedures to account for complex sampling design. We computed weighted prevalence estimates and 95% CIs for all measures. We did not report results with unstable estimates (ie, unweighted denominators <50 or a relative standard error >30%). We used χ^2^ tests to assess the differences among groups with *P* < .05 considered significant. This activity was reviewed and deemed not research by the Centers for Disease Control and Prevention (CDC) and was conducted consistent with applicable federal law and CDC policy.

## Results

### Youth characteristics

In 2024, 5.9% of students reported current e-cigarette use. Of these, 74.6% were high school students (Table 1). Current e-cigarette use was associated with school level, race and ethnicity, grades in school, and symptoms of depression and anxiety. Moderate-to-severe symptoms of depression and anxiety were reported by 42.1% of students who currently used e-cigarettes compared with 21.0% of students who did not use e-cigarettes (*P* < .001). Among youth who currently used e-cigarettes, symptom severity was associated with sex and grades in school (Table 2).

**Table 1:** Characteristics of Youth (Grades 6–12), by E-Cigarette Use Status, National Youth Tobacco Survey, United States, 2024

Characteristic	Current e-cigarette use,^a^ weighted % (95% CI)	Former or never e-cigarette use,^b^ weighted % (95% CI)	*P* value^c^
**Overall^d^ (n = 29,501)**	**5.9 (5.3–6.6)**	**94.1 (93.4–94.7)**	—
**School level (n = 29,347)**			
Middle school (6th–8th grade)	25.4 (20.5–31.2)	44.2 (39.3–49.2)	<.001
High school (9th–12th grade)	74.6 (68.8–79.5)	55.8 (50.8–60.7)	
**Sex (n = 29,335)**			
Male	49.9 (46.4–53.5)	51.6 (50.5–52.6)	.35
Female	50.1 (46.5–53.6)	48.4 (47.4–49.5)	
**Race and ethnicity^e^ (n = 28,711)**			
American Indian or Alaska Native	1.8 (1.3–2.5)	0.9 (0.7–1.0)	<.001
Asian	2.1 (1.3–3.4)	5.6 (4.6–6.8)	
Black or African American	17.0 (12.4–23.0)	14.4 (11.4–18.1)	
Native Hawaiian or Pacific Islander	—f	0.3 (0.2–0.4)	
White	44.7 (37.4–52.3)	45.9 (40.8–51.2)	
Hispanic or Latino	29.1 (23.6–35.3)	28.5 (24.0–33.4)	
Multiracial	4.8 (3.8–6.1)	4.4 (3.9–4.8)	
**Grades in school (n = 23,743)**			
Mostly As	32.5 (28.8–36.5)	49.5 (47.1–51.8)	<.001
Mostly Bs	28.6 (25.2–32.2)	33.0 (31.7–34.4)	
Mostly Cs	23.2 (20.3–26.3)	12.3 (11.3–13.4)	
Mostly Ds	9.6 (7.6–11.9)	3.1 (2.7–3.6)	
Mostly Fs	6.2 (4.5–8.5)	2.0 (1.8–2.4)	
**Symptoms of depression and anxiety^g^ (n = 24,782)**			
None	37.2 (33.5–41.0)	57.1 (55.9–58.3)	<.001
Mild	20.7 (18.0–23.7)	21.8 (21.1–22.5)	
Moderate	19.3 (16.9–22.0)	11.8 (11.3–12.4)	
Severe	22.8 (20.2–25.6)	9.2 (8.7–9.9)	
**Symptoms of depression and anxiety (dichotomized)^h^**			
None-to-mild	57.9 (54.6–61.1)	78.9 (78.0–79.8)	<.001
Moderate-to-severe	42.1 (38.9–45.4)	21.0 (20.2–22.0)	

a Current use was defined as use on ≥1 day during the previous 30 days.

b Former use was defined as ever using an e-cigarette but not in the last 30 days. Never use was defined as not ever using e-cigarettes.

c Chi-square tests were used to assess the differences among groups, with *P* < .05 considered significant. Comparisons were based on available responses excluding missing data.

d Top row represents row percentages (ie, prevalence estimates). Subsequent rows represent column percentages (ie, within group proportions).

e Hispanic or Latino (Hispanic) people might be of any race; all races listed are non-Hispanic.

f Dash indicates that data are statistically unreliable because sample size was <50 or relative standard error was >0.3.

g Symptoms of depression and anxiety were assessed by using the 4-item Patient Health Questionnaire (PHQ-4) (7) that asks youth to assess the frequency (not at all, several days, more than half the days, nearly every day) of 4 characteristics (little interest, feeling down, nervous, worried) experienced during the past 2 weeks. A composite score based on all 4 questions was created (none [0–2], mild [3–5], moderate [6–8], severe [9–12]).

h The PHQ-4 composite score was dichotomized to no-to-mild (score, 0–5) and moderate-to-severe (score, 6–12).

**Table 2:** Characteristics of Youth (Grades 6–12) Reporting Current (Past 30-Day) Use^a^ of E-Cigarettes, by Symptoms of Depression and Anxiety,^b^ National Youth Tobacco Survey, United States, 2024

Characteristic	Symptoms of depression and anxiety		*P* value^c^
	None-to-mild, weighted % (95% CI)	Moderate-to-severe, weighted % (95% CI)	
**Overall^d^ (n = 1,219)**	**57.9 (54.6–61.1)**	**42.1 (38.9–45.4)**	** —**
**School level (n = 1,213)**			
Middle school (6th–8th grade)	21.0 (15.9–27.4)	23.9 (18.3–30.6)	.33
High school (9th–12th grade)	79.0 (72.6–84.1)	76.1 (69.4–81.7)	
**Sex (n = 1,215)**			
Male	53.9 (48.9–58.9)	38.1 (33.0–43.4)	<.001
Female	46.1 (41.1–51.1)	61.9 (56.6–67.0)	
**Race and ethnicity^e^ (n = 1,207)**			
American Indian or Alaska Native	2.2 (1.3–3.8)	0.9 (0.5–1.6)	.12
Asian	—f	—f	
Black or African American	16.3 (10.9–23.8)	12.5 (8.0–19.2)	
Native Hawaiian or Pacific Islander	—f	—f	
White	48.5 (40.1–56.9)	47.5 (39.6–55.5)	
Hispanic or Latino	26.0 (19.2–34.2)	30.8 (24.8–37.6)	
Multiracial	4.2 (2.7–6.4)	6.1 (4.2–8.9)	
**Grades in school (n = 1,115)**			
Mostly As	38.0 (32.3–44.0)	26.9 (22.1–32.2)	<.001
Mostly Bs	28.6 (24.2–33.3)	27.9 (23.1–33.1)	
Mostly Cs	22.8 (18.5–27.7)	24.4 (20.1–29.4)	
Mostly Ds	6.8 (4.7–9.6)	12.3 (9.1–16.5)	
Mostly Fs	3.9 (2.5–6.1)	8.5 (5.6–12.7)	

a Current use was defined as use on ≥1 day during the previous 30 days.

b Symptoms of depression and anxiety were assessed using the 4-item Patient Health Questionnaire (7) that asks youth to assess the frequency (not at all, several days, more than half the days, nearly every day) of 4 characteristics (little interest, feeling down, nervous, worried) experienced during the past 2 weeks. A composite score based on all 4 questions was dichotomized (score of 0–5, no-to-mild symptoms; score of 6–12, moderate-to-severe symptoms).

c Chi-square tests were used to assess the differences among groups, with *P* < .05 considered significant. Comparisons based on available responses excluding missing data.

d Top row represents row percentages. Subsequent rows represent column percentages.

e Hispanic or Latino (Hispanic) people might be of any race; all races listed are non-Hispanic.

f Dash indicates that data are statistically unreliable because sample size was <50 or relative standard error was >0.3.

### E-cigarette use behaviors

Among students with current e-cigarette use, some use behaviors differed by symptoms of depression and anxiety (Table 3). A higher percentage of youth with moderate-to-severe symptoms (vs no-to-mild symptoms) wanted to use a tobacco product within 30 minutes of waking (28.2% vs 15.6%, *P* < .001) and reported having strong cravings to use tobacco (37.6% vs 22.4%, *P* < .001). More than 90% of youth reported using flavored e-cigarettes and more than one third reported co-use of other tobacco products, irrespective of symptoms of poor mental health; no significant between-group differences were found for these measures.

**Table 3:** E-Cigarette Use Behaviors of Youth (Grades 6–12) Reporting Current (Past 30-Day) Use^a^ of E-Cigarettes, by Symptoms of Depression and Anxiety,^b^ National Youth Tobacco Survey, United States, 2024

E-cigarette use behavior	Overall, weighted % (95% CI)	Symptoms of depression and anxiety		*P* value^c^
		None-to-mild, weighted % (95% CI)	Moderate-to-severe, weighted % (95% CI)	
**Frequency of use,^d^ no. of days (n = 1,219)**				
1–5	46.1 (41.7–50.4)	49.0 (44.2–53.8)	42.1 (35.5–49.0)	.01
6–19	17.9 (15.4–20.4)	19.3 (16.0–23.1)	16.0 (13.1–19.4)	
20–30	36.0 (31.3–40.8)	31.7 (27.1–36.7)	41.9 (35.4–48.8)	
**Time to first use after waking^e^ (n = 1,210)**				
Within 30 min	20.9 (18.1–23.8)	15.6 (12.9–18.8)	28.2 (23.7–33.3)	<.001
Not within 30 min	79.1 (76.2–81.9)	84.4 (81.2–87.1)	71.8 (66.7–76.3)	
**Strong craving for tobacco product^f^ (n = 1,215)**				
Yes	28.8 (25.9–31.6)	22.4 (19.0–26.2)	37.6 (32.9–42.5)	<.001
No	71.2 (68.4–74.1)	77.6 (73.8–81.0)	62.4 (57.5–67.1)	
**Flavored e-cigarette use^g^ (n = 1,219)**				
Yes	92.6 (90.9–94.3)	92.7 (90.1–94.7)	92.5 (89.0–94.9)	.97
No	5.6 (4.0–7.2)	5.6 (3.8–8.1)	5.6 (3.6–8.8)	
Unknown	—h	—h	—h	
**Co-use of other tobacco products^i^ (n = 1,219)**				
Yes	38.4 (35.1–41.7)	35.7 (30.8–40.8)	42.2 (37.9–46.6)	.06
No	61.6 (58.3–64.9)	64.3 (59.2–69.2)	57.8 (53.4–62.1)	
**Seriously thinking about quitting^j^ (n = 1,176)**				
Yes	73.8 (70.3–77.4)	77.7 (73.2–81.7)	68.7 (63.3–73.6)	.004
No	26.2 (22.6–29.7)	22.3 (18.3–26.8)	31.3 (26.4–36.7)	
**Attempted to quit (during the past 12 months)^k^ (n = 1,176)**				
Yes	69.4 (66.5–72.3)	71.0 (67.3–74.5)	67.2 (61.2–72.7)	.29
No	30.6 (27.7–33.5)	29.0 (25.5–32.7)	32.8 (27.3–38.8)	
**Resources used in quit attempt^l^ (n = 830)**				
Did not use any resources	58.5 (54.5–62.5)	60.6 (54.7–66.2)	55.6 (49.5–61.5)	.26
Help or advice from an adult (parent/caregiver or teacher/coach)^m^	9.7 (7.4–12.1)	7.6 (5.5–10.4)	12.8 (8.8–18.3)	.04
Help or advice from a friend or peer	13.2 (10.2–16.2)	10.7 (7.8–14.6)	16.8 (11.7–23.5)	.07
Medical treatment or counseling^n^	7.5 (5.1–9.8)	7.0 (4.5–10.8)	8.1 (5.1–12.8)	.66
Quitline, internet, app, or texting program^o^	12.5 (10.1–15.0)	8.9 (6.2–12.7)	17.7 (14.2–21.9)	.001
Other	11.5 (9.0–14.1)	13.8 (10.7–17.6)	8.3 (5.2–13.2)	.06

a Current use was defined as use on ≥1 day during the previous 30 days.

b Symptoms of depression and anxiety were assessed using the 4-item Patient Health Questionnaire (7) that asks youth to assess the frequency (not at all, several days, more than half the days, nearly every day) of 4 characteristics (little interest, feeling down, nervous, worried) experienced during the past 2 weeks. A composite score based on all 4 questions was dichotomized (score of 0–5, no-to-mild symptoms; score of 6–12, moderate-to-severe symptoms).

c Chi-square tests were used to assess the differences among groups, with *P* < .05 considered significant. Comparisons based on available responses excluding missing data.

d Frequency of use was determined based on the question, “During the past 30 days, on how many days did you use e-cigarettes?”

e Time to first use after waking was determined from the question, “How soon after you wake up do you want to use a tobacco product of any kind?”

f Strong craving for a tobacco product was assessed by the question, “During the past 30 days, have you had a strong craving or felt like you really needed to use a tobacco product of any kind?”

g Flavored e-cigarette use was defined as using any flavor other than “tobacco-flavored” or “unflavored” (yes). Report of using only “tobacco-flavored” or “unflavored” was defined as unflavored product use (no). Those who provided no valid responses were classified as “unknown.”

h Dash indicates that data are statistically unreliable because sample size was <50 or relative standard error was >0.3.

i Co-use is defined as current use of e-cigarettes and current use (past 30 days) of 1 or more of the following tobacco products: cigars, cigarettes, smokeless tobacco (chewing tobacco, snuff, dip, or snus), hookahs, nicotine pouches, heated tobacco products, pipe tobacco, bidis (small brown cigarettes wrapped in a leaf), or other oral nicotine products.

j Seriously thinking about quitting was determined by the question, “Are you seriously thinking about quitting e-cigarettes?”

k Attempted to quit was determined by the question, “During the past 12 months, how many times have you stopped using e-cigarettes for 1 day or longer because you were trying to quit using e-cigarettes for good?” Response was coded as yes for at least 1 time and coded as no for 0 times.

l Resources used in quit attempts was determined for those who tried to quit based on the question, “When you tried to quit using e-cigarettes, did you use any of the following?” Each resource type was assessed as an individual dichotomous variable; only yes answers are displayed. Chi-square tests were performed for each quitting resource individually (yes vs no).

m Two response options were combined to create 1 dichotomized variable: “Help or advice from a parent or caregiver” and “Help or advice from a teacher or coach.”

n Two response options were combined to create 1 dichotomized variable: “Help, advice, or counseling from a doctor or health care provider” and “Treatment from a hospital, medical center, or some other facility.”

o Three response options were combined to create 1 dichotomized variable: “Help or advice from the internet,” “a mobile app or texting program,” or “a telephone helpline or quitline.”

A lower percentage of youth with moderate-to-severe symptoms (vs no-to-mild symptoms) reported seriously thinking about quitting (68.7% vs 77.7%, *P* = .004). Most youth from both groups reported attempting to quit e-cigarette use in the past 12 months (69.4% overall), but more than half who tried to quit did not use any quitting resources (58.5%); there were no significant between-group differences for these measures. The most commonly used quitting resources among youth with moderate-to-severe symptoms of depression and anxiety were a quitline, the internet, a mobile app, or a texting program (17.7%), help or advice from a friend or peer (16.8%), and help or advice from a parent, caregiver, teacher, or coach (12.8%).

### Reasons for e-cigarette use

The most common reasons for first e-cigarette use among youth currently using e-cigarettes were “A friend used them” and “I was curious about them” (Table 4). This was true both for youth with moderate-to-severe symptoms of depression and anxiety and those with no-to-mild symptoms. Among youth who reported moderate-to-severe symptoms, the most common reasons for current use were “Because I feel anxious, stressed, or depressed” and “To get a high or buzz from nicotine.” Among youth with no-to-mild symptoms, the most common reasons for current use were “A friend uses them” and “To get a high or buzz from nicotine.” Youth who reported moderate-to-severe symptoms (vs no-to-mild symptoms) more frequently reported “feeling anxious, stressed, or depressed” as a reason for first use (41.8% vs 18.4%, *P* < .001) and current use (51.0% vs 25.2%, *P* < .001).

**Table 4:** Top 5 Reasons for First and Current E-Cigarette Use Among Youth (Grades 6–12) Reporting Current (Past 30-Day) Use^a^ of E-Cigarettes, by Symptoms of Depression and Anxiety,^b^ National Youth Tobacco Survey, United States, 2024

Reason for use	Symptoms of depression and anxiety		*P* value^c^
	None-to-mild, % (95% CI)	Moderate-to-severe, % (95% CI)	
**Reason for first e-cigarette use**			
A friend used them	53.2 (47.8–58.5)	54.1 (48.9–59.3)	.80
I was curious about them	39.1 (34.8–43.4)	42.9 (37.1–48.7)	.32
I was feeling anxious, stressed, or depressed	18.4 (15.0–21.8)	41.8 (36.6–47.0)	<.001
To get a high or buzz from nicotine	24.8 (21.4–28.3)	32.2 (27.2–37.3)	.02
A family member used them	23.2 (19.5–27.0)	31.6 (26.3–37.0)	.02
**Reason for current e-cigarette use**			
Because I feel anxious, stressed, or depressed	25.2 (21.3–29.2)	51.0 (45.7–56.3)	<.001
To get a high or buzz from nicotine	29.3 (24.7–34.0)	35.8 (30.3–41.2)	.04
A friend uses them	29.4 (24.6–34.3)	26.4 (22.2–30.7)	.30
I can use them to do tricks	16.6 (13.3–20.0)	20.1 (16.2–24.1)	.23
They are available in flavors	—d	15.5 (11.4–19.6)	—e
Other	15.6 (12.0–19.2)	—d	—e

a Current use was defined as use on ≥1 day during the previous 30 days.

b Symptoms of depression and anxiety were assessed using the 4-item Patient Health Questionnaire (7) that asks youth to assess the frequency (not at all, several days, more than half the days, nearly every day) of 4 characteristics (little interest, feeling down, nervous, worried) experienced during the past 2 weeks. A composite score based on all 4 questions was dichotomized (score of 0–5, no-to-mild symptoms; score of 6–12, moderate-to-severe symptoms).

c Chi-square tests were used to assess the differences among groups, with *P* < .05 considered significant. Comparisons based on available responses excluding missing data.

d Dash indicates reason was not in the top 5 for this group.

e Chi-square test was not performed because reason was not in the top 5 for one of the groups.

## Discussion

In 2024, approximately 2 in 5 US middle and high school students who currently used e-cigarettes reported moderate-to-severe symptoms of depression and anxiety (symptoms of poor mental health) compared with 1 in 5 students who did not use e-cigarettes. This finding is consistent with previous literature describing an association between e-cigarette use and poor mental health among youth (3,4). Youth mental health problems are a persistent and growing public health concern (8,9). Poor mental health among youth is associated with poor physical health, challenges with school, and difficulties with decision making (10–12).

While the most commonly reported reasons for youth to try e-cigarettes were social influences and curiosity, a greater percentage of youth with moderate-to-severe (vs no-to-mild) symptoms of depression and anxiety reported starting e-cigarette use because they felt stressed, anxious, or depressed. This was also the leading reason for current e-cigarette use in this group and the third most common reason for current use among youth with no-to-mild symptoms. This suggests that youth may be using e-cigarettes to try to cope with symptoms of poor mental health. In addition, nicotine addiction and withdrawal can contribute to or worsen feelings of anxiety and depression (13). The developing adolescent brain is uniquely susceptible to nicotine, and youth can start showing signs of addiction quickly, sometimes before the onset of regular or daily tobacco product use (2). Nicotine addiction can itself be a source of stress (14).

The results of this study suggest that youth, particularly those with poor mental health, may need support learning healthy coping skills to help prevent initiation of e-cigarette use; those who use e-cigarettes may need similar support to encourage cessation. Such skills might include emotion recognition and management, positive stress management techniques, self-care routines (eg, physical activity, eating a balanced diet, getting enough sleep), and building a support system of adults and peers who are supportive and encouraging (8). CDC’s school action guide, *Promoting Mental Health and Well-Being in Schools*, highlights 6 evidence-based strategies to improve youth mental health including increasing coping skills and strategies and providing psychosocial skills training and cognitive behavioral interventions (15). Youth with symptoms of poor mental health may additionally need further behavioral health assessment and treatment as appropriate (8,16).

The findings of this report suggest that youth who use e-cigarettes and report symptoms of poor mental health may need additional cessation treatment and support and encouragement to quit. These youth exhibit a higher prevalence of nicotine dependence symptoms than their counterparts (ie, youth who use e-cigarettes and do not report symptoms of poor mental health), and a lower percentage express a desire to quit. Even so, this study is consistent with previous reports that most youth who use e-cigarettes want to quit, most have tried to quit, and more than half who tried to quit did so without using any quitting resources (17,18). Quitting resources tailored for youth are available online (eg, www.teen.smokefree.gov), over the telephone (eg, 1–800-QUIT-NOW), via text programs (eg, SmokefreeTXT for teens: text QUIT to 47848), and via smartphone apps (eg, quitSTART). This study found that these types of quitting resources are the types most commonly used by youth reporting symptoms of poor mental health, suggesting that such resources may benefit from inclusion of content about healthy coping skills and other resources to support mental health. Quitting resources may also benefit from addressing co-use of multiple tobacco products; data from this study show that more than one-third of youth who use e-cigarettes also use another tobacco product.

Trusted adults play an important role in guiding youth and connecting them to resources for tobacco cessation and mental health (2,8). Our findings indicate that youth who use quitting resources sometimes turn to parents or educators for support in quitting e-cigarette use, suggesting that these adults are an important resource for youth. Education and health care settings are critical spaces to support youth mental health and promote tobacco use prevention and cessation. School connectedness, or a sense of belonging at school, is associated with a reduced risk of smoking initiation and improved mental health (19,20). Educators can create and maintain safe and supportive school environments that are tobacco-free, incorporate mental health and tobacco prevention into adolescent health programs, facilitate access to treatment to support students to quit using tobacco products rather than focusing solely on punitive action, and empower youth to resist peer pressure and make positive decisions (2,8,21,22). These supportive actions may be particularly valuable for youth with lower academic performance; data in this study suggest a potential inverse relationship between academic performance and symptoms of poor mental health among youth with current e-cigarette use. Health care providers can screen all teenagers for mental health concerns and tobacco product use, provide youth with education and prevention messages, and connect patients to quitting and behavioral health resources (16,23). Comprehensive population-level approaches at state and community levels, including public education media campaigns and tobacco retail environment strategies (eg, increasing tobacco product price, preventing access to e-cigarettes by youth, requiring tobacco retailer licensure, prohibiting the sale of flavored tobacco products), support tobacco use prevention and cessation (2,21). Similarly, evidence-based programs that promote healthy development, support youth and their families, and increase youth resilience can support youth mental health promotion and tobacco use prevention and cessation (8).

The findings in this study are subject to some limitations. First, the cross-sectional data of NYTS do not allow for causal inferences; therefore, we cannot infer that symptoms of poor mental health cause e-cigarette use, or vice-versa. Future studies could consider using longitudinal data to investigate the directionality of associations between e-cigarette use, cessation, and mental health outcomes. Second, the overall survey response rate was 33%; per NYTS methods, we made adjustments to the survey weights to reduce nonresponse bias (24). Third, bias from nonresponse to PHQ-4 questions may be present. While literature suggests that there is no consistent relationship between mental health status and response to survey questions about mental health (25), our findings may be subject to bias if PHQ-4 nonresponders were differentially likely to experience symptoms of depression and anxiety. Fourth, findings are not generalizable to youth who are home-schooled, have dropped out of school, are in detention centers, or are enrolled in alternative schools. Fifth, self-reported data are subject to recall and social desirability bias that can lead to over- or underreporting. Lastly, while the PHQ-4 is a practical, reliable, and valid screening tool, it is not a diagnostic tool, precluding determination of clinical diagnoses of depression and anxiety (6,7).

Symptoms of poor mental health are more commonly reported among youth who currently use e-cigarettes compared with those who do not. Youth with symptoms of depression and anxiety may need additional or tailored support to prevent them from starting e-cigarette use and to help them quit if they have already started. Integrating mental health support into comprehensive approaches to tobacco use prevention and cessation, paired with strengthening the foundations of healthy communities for youth, may reduce youth e-cigarette use.
